# Prognostic and Inflammatory Differences Between Upper and Mid–Lower Rectal Cancers in Non-Metastatic Stage II–II Disease

**DOI:** 10.3390/curroncol32040227

**Published:** 2025-04-12

**Authors:** Fırat Mülküt, Cem Batuhan Ofluoğlu, Mustafa Kağan Başdoğan, İsa Caner Aydın, Akif Doğan, İsmail Ege Subaşı

**Affiliations:** 1 Department of General Surgery, Sancaktepe Şehit Prof. Dr. İlhan Varank Training and Research Hospital, University of Health Sciences, 34785 Istanbul, Turkey; m.basdogan@saglik.gov.tr; 2 Department of Gastrointestinal Surgery, Sancaktepe Şehit Prof. Dr. İlhan Varank Training and Research Hospital, University of Health Sciences, 34785 Istanbul, Turkey; cem.ofluoglu@saglik.gov.tr (C.B.O.); ismailege.subasi@saglik.gov.tr (İ.E.S.); 3 Department of Gastrointestinal Surgery, Ministry of Health Zonguldak Atatürk State Hospital, 67030 Zonguldak, Turkey; isacaner.aydin@saglik.gov.tr; 4 Department of Medical Oncology, Sancaktepe Şehit Prof. Dr. İlhan Varank Training and Research Hospital, University of Health Sciences, 34785 Istanbul, Turkey; akif.dogan1@saglik.gov.tr

**Keywords:** rectal cancer, upper rectal cancer, mid–lower rectal cancer, prognostic factors, inflammatory markers, survival analysis

## Abstract

**Background:** This study aimed to compare the clinical, pathological, and biochemical characteristics of upper rectal cancer (URC) and mid–lower rectal cancer (MLRC) in stage II and III non-metastatic rectal cancer and to identify distinct prognostic factors influencing survival and recurrence. **Material and Methods:** This retrospective cohort study included 100 patients with stage II and III non-metastatic rectal adenocarcinoma who underwent neoadjuvant chemoradiotherapy (nCRT) followed by curative-intent surgery between 2021 and 2024. Patients were categorized into URC (n = 53) and MLRC (n = 47) groups. Parameters analyzed included demographic factors, ASA score, surgical characteristics, pathological features (tumor stage, lymph node involvement, lymphovascular invasion (LVI), perineural invasion (PNI), tumor budding, tumor regression grade (TRG)), and biochemical markers (carcinoembryonic antigen (CEA), carbohydrate antigen 19-9 (CA19-9), white blood cell (WBC) count, neutrophil count, platelet count (PLT), and C-reactive protein (CRP)). One-year overall survival (OS) and disease-free survival (DFS) were analyzed using Kaplan–Meier survival curves, and Cox regression models identified independent prognostic factors. **Results:** Preoperative CEA levels were higher in MLRC (*p* = 0.05), whereas WBC count (*p* = 0.01), neutrophil count (*p* = 0.02), PLT (*p* = 0.01), and CRP levels (*p* = 0.01) were higher in URC. Pathological analysis revealed higher LVI (*p* = 0.04), PNI (*p* = 0.04), and tumor budding (*p* = 0.03) in MLRC. At one-year follow-up, OS rates were 82.1% (URC) vs. 80.3% (MLRC) (*p* = 0.85), and DFS rates were 78.6% (URC) vs. 73.4% (MLRC) (*p* = 0.72). Multivariate Cox regression analysis identified age (HR: 1.04, *p* = 0.03), ASA score (HR: 1.22, *p* = 0.01), CRP (HR: 1.18, *p* < 0.001), preoperative CEA (HR: 1.12, *p* = 0.02), preoperative CA19-9 (HR: 1.09, *p* = 0.03), LVI (HR: 1.42, *p* < 0.001), PNI (HR: 1.35, *p* = 0.02), and tumor budding (HR: 1.28, *p* = 0.03) as independent prognostic factors for OS. Similar trends were observed for DFS, with T stage (HR: 1.35, *p* = 0.01) and tumor size (HR: 1.22, *p* = 0.01) also being found significant. **Conclusions:** Inflammatory markers, tumor burden indicators (LVI, PNI, budding, tumor size, T stage), and preoperative CEA/CA19-9 were identified as significant predictors, suggesting a risk-adapted approach to rectal cancer treatment.

## 1. Introduction

Rectal cancer represents approximately one-third of all colorectal malignancies, with an estimated incidence of 20 new cases per 100,000 individuals annually in Europe [1]. Despite advancements in multimodal treatment strategies, significant heterogeneity exists in clinical outcomes, particularly concerning tumor location within the rectum. Anatomical and oncological distinctions between upper rectal cancer (URC) and mid–lower rectal cancer (MLRC) influence tumor behavior, treatment response, and prognosis.

The rectum is conventionally divided into upper (10–15 cm from the anal verge), middle (5–10 cm), and lower (0–5 cm) segments, and the likelihood of achieving complete surgical resection URC is often considered biologically closer to colon cancer and has a lower risk of local recurrence. MLRC presents greater surgical challenges due to the confines of the pelvis and a higher propensity for circumferential margin involvement. Given these differences, questions remain regarding the necessity of neoadjuvant chemoradiotherapy (nCRT) for URC and whether treatment strategies should be adapted based on tumor location [2].

Several studies suggest that URC exhibits lower local recurrence rates and improved disease-free survival (DFS) compared to MLRC, raising concerns about the potential overtreatment of URC with aggressive nCRT protocols. In contrast, MLRC—especially distal tumors—is associated with higher rates of circumferential resection margin (CRM) positivity, greater reliance on nCRT, and increased local recurrence [3]. These variations necessitate a more tailored approach to treatment, particularly regarding the role of neoadjuvant therapy in URC.

Although total mesorectal excision (TME) remains the gold standard for curative surgery, emerging evidence suggests that partial mesorectal excision (PME) may be sufficient for URC, potentially reducing morbidity without compromising oncological outcomes. However, the lack of standardized guidelines for treating URC differently from MLRC creates uncertainty in clinical practice.

Despite notable advances in rectal cancer treatment, the extent to which treatment strategies should be individualized based on tumor location remains a subject of debate. Emerging evidence highlights key biological distinctions between URC and MLRC, including disparities in systemic inflammatory responses and tumor aggressiveness. Inflammatory markers, such as C-reactive protein (CRP), white blood cell (WBC) count, and neutrophil count, have been repeatedly associated with poorer prognosis in colorectal cancer [4,5]; however, direct comparisons of these markers between URC and MLRC—and their interplay with histopathological features—remain scarce.

A more nuanced, risk-adaptive strategy that extends beyond pure anatomical localization may thus be warranted. Particularly, data suggesting higher inflammatory marker levels in URC versus more aggressive pathological signatures (lymphovascular invasion, perineural invasion, tumor budding) in MLRC could inform both prognostic evaluation and therapeutic decision-making [6,7]. To our knowledge, few studies have systematically explored how these divergent biological profiles influence outcomes following standardized nCRT. The current study therefore aims to fill this gap by comparing URC and MLRC within a single nCRT-treated cohort, focusing on the correlations between inflammatory markers, tumor biology, and prognostic endpoints.

To our knowledge, this is one of the first studies to systematically evaluate the prognostic impact of inflammatory biomarkers, pathological risk features, and short-term outcomes between upper and mid–lower rectal cancer patients treated with uniform nCRT and curative surgery. This stratified comparison highlights the biological heterogeneity between anatomical subgroups and addresses an underexplored gap in the current literature.

This study aimed to compare the clinical, pathological, and biochemical characteristics of URC and MLRC in patients with stage II and III non-metastatic rectal cancer undergoing nCRT followed by curative-intent surgery. By identifying distinct prognostic factors associated with survival and disease recurrence, this study aimed to determine whether tumor location should be integrated into risk stratification models and treatment decision-making to optimize oncological outcomes while minimizing unnecessary treatment-related morbidity.

## 2. Material and Methods

### 2.1. Study Design and Population

This retrospective cohort study was conducted at a tertiary care center and included patients diagnosed with rectum cancer between 2021 and 2024.

Patient selection was based on histopathologically confirmed rectal adenocarcinoma (≤15 cm from the anal verge), as confirmed through imaging or surgical findings. Only patients with non-metastatic (M0) disease at diagnosis, as verified through radiological and pathological evaluation, were included. Stage classification was performed according to the AJCC TNM staging system. Eligible patients were those who underwent primary tumor resection with curative intent and had complete clinical, surgical, and pathological data available for analysis. All patients received standardized long-course nCRT consistent with internationally accepted guidelines.

Patients were excluded if they had incomplete staging, missing essential clinical data, or were lost to follow-up before definitive treatment or during postoperative surveillance. Additional exclusion criteria included the presence of synchronous metastatic disease at diagnosis, a history of previous malignancies within the past five years, recurrent rectal cancer, or cases where surgery was incomplete or palliative rather than curative.

### 2.2. Neoadjuvant Chemoradiotherapy and Surgical Timing

All patients received nCRT in accordance with international guidelines prior to definitive surgical intervention [8]. Indications for nCRT in stage II rectal cancer (T3N0) specifically include at least one high-risk feature, such as threatened or involved CRM, extramural vascular invasion (EMVI), poor differentiation, or deep submucosal (sm3) infiltration. Patients meeting any of these criteria are deemed suitable for long-course nCRT, consistent with NCCN and ESMO guidelines [8,9]. Surgical technique was determined according to tumor location and mesorectal involvement. PME was reserved for upper rectal lesions (≥10 cm from the anal verge) without evidence of mesorectal fascia compromise, whereas TME was mandated for mid–lower rectal tumors due to their higher propensity for circumferential margin involvement.

The RT protocol consisted of a total dose of 50.4 Gy, administered in daily fractions of 1.8 Gy, five days per week, over 5–6 weeks (28 fractions). Concurrent chemotherapy was delivered using either oral capecitabine (825 mg/m² twice daily) or continuous infusion 5-fluorouracil (5-FU, 225 mg/m^2^/day) throughout the RT course. Capecitabine was administered 1–2 h before RT sessions and at 12 h intervals on treatment days, whereas 5-FU was continuously infused for the entire RT duration. Following the completion of nCRT, curative-intent surgery was scheduled 6–8 weeks later to optimize tumor regression and facilitate potential downstaging. Adjuvant chemotherapy was administered only to high-risk patients based on postoperative pathological assessment, while patients without these criteria were excluded from the study. Eligible patients received either CAPOX (XELOX) or FOLFOX regimens. The CAPOX regimen included capecitabine (1000 mg/m² orally, twice daily on days 1–14) and oxaliplatin (130 mg/m² IV on day 1), repeated every 3 weeks for 6–8 cycles. The FOLFOX regimen consisted of oxaliplatin (85 mg/m² IV on day 1), leucovorin (400 mg/m² IV on day 1), and 5-FU (400 mg/m² IV bolus followed by 2400 mg/m² continuous infusion over 46 h), repeated every 2 weeks for 12 cycles. The total duration of adjuvant chemotherapy was 6 months, with regimen selection based on patient performance status, toxicity profile, and multidisciplinary oncology board recommendations.

### 2.3. Data Collection

Clinical, surgical, pathological, and biochemical data were extracted from institutional electronic medical records. Demographic variables included patient age, sex, and comorbidities, along with the ASA score. Surgical variables included operative approach (laparoscopic or open), type of anastomosis, presence of a protective ostomy, duration of surgery, and length of hospital stay. Pathological data encompassed tumor stage, lymph node involvement, tumor size, number of retrieved lymph nodes, presence of lymphovascular invasion (LVI), perineural invasion (PNI), tumor budding, and histopathological tumor regression grade (TRG) following nCRT. Biochemical markers included preoperative and postoperative carcinoembryonic antigen (CEA) and carbohydrate antigen 19-9 (CA19-9) levels, white blood cell count, neutrophil count, platelet count, hemoglobin, CRP, and albumin levels. Oncological outcomes assessed included tumor response to nCRT, local recurrence, distant metastasis, and one-year mortality (exitus).

#### 2.3.1. Ethical Consideration

This study was approved by the institutional ethics committee of Sancaktepe Şehit Prof. Dr. İlhan Varank Training and Research Hospital Scientific Research Ethics Committee on 22 January 2025 (Approval Code: Dosya No: 27), and all procedures were conducted in compliance with the Declaration of Helsinki.

#### 2.3.2. Statistical Analyses

Statistical analyses were performed using SPSS version 24.0. Continuous variables were tested for normality using the Kolmogorov–Smirnov test and analyzed using either the independent *t*-test or the Mann–Whitney U test, depending on the distribution of data. Categorical variables were compared using the Chi-square test or Fisher’s exact test. One-year overall survival (OS) and disease-free survival (DFS) were analyzed using Kaplan–Meier survival curves, and group comparisons were performed using the log-rank test. Cox proportional hazards regression models were constructed to identify independent predictors of OS and DFS, adjusting for age, tumor characteristics, pathological features, and inflammatory markers. Hazard ratios (HR) and 95% confidence intervals (CI) were reported for all significant variables. The proportional hazards (PH) assumption using Martingale residual plots and time-dependent covariates were assessed, finding no violations of the PH assumption. Multicollinearity was evaluated through variance inflation factors (VIF) and tolerance statistics for each covariate; all VIF values were below 2, indicating no significant multicollinearity issues. For select comparisons approaching the conventional significance threshold (*p* ~ 0.05), we performed sensitivity analyses by excluding patients with incomplete neoadjuvant treatment or insufficient follow-up (<6 months). Repeating the Cox regression under these conditions confirmed similar effect sizes and trends. Statistical significance was set at *p* < 0.05.

## 3. Results

A total of 100 patients diagnosed with non-metastatic stage II and III rectal cancer were included in the study. Among them, 53 (55.0%) had tumors located in the upper rectum, while 47 (45.0%) had tumors in the mid–lower rectum. There were no statistically significant differences between the upper rectum and mid–lower rectum groups in terms of age (*p* = 0.36), gender distribution (*p* = 0.89), ASA score (*p* = 0.10), hospital stay duration (*p* = 0.40), laparoscopic surgery rate (*p* = 0.08), protective ostomy rate (*p* = 0.15), and circular anastomosis rate (*p* = 0.80).

Preoperative CEA levels showed a borderline statistical significance (*p* = 0.05), with higher levels observed in the mid–lower rectum group compared to the upper rectum group. WBC count (*p* = 0.01), neutrophil count (*p* = 0.02), platelet count (*p* = 0.01), and CRP levels (*p* = 0.01) were significantly higher in the upper rectum group compared to the mid–lower rectum group. Additionally, the number of removed lymph nodes was significantly higher in the upper rectum group (*p* = 0.00). Regarding histopathological characteristics, LVI (*p* = 0.04), PNI (*p* = 0.04), and tumor budding (*p* = 0.03) were significantly more frequent in the mid–lower rectum group (Table 1).

The one-year OS rates were 82.1% (95% CI: 72.3–91.9) in the upper rectum group and 80.3% (95% CI: 70.1–90.5) in the mid–lower rectum group, with no statistically significant difference between the two groups (*p* = 0.85). The one-year DFS rates were 78.6% (95% CI: 67.8–89.4) in the upper rectum group and 73.4% (95% CI: 62.1–84.7) in the mid–lower rectum group, and this difference was not statistically significant (*p* = 0.72) (Table 2).

In the multivariate logistic regression analysis for OS, age (HR: 1.04; 95% CI: 1.01–1.08, *p* = 0.03) and ASA score (HR: 1.22; 95% CI: 1.10–1.38, *p* = 0.01) were significant predictors of mortality at one year. Elevated WBC count (HR: 1.03; 95% CI: 1.01–1.06, *p* = 0.04) and CRP levels (HR: 1.18; 95% CI: 1.08–1.29, *p* < 0.001) were also independently associated with poorer survival outcomes. Preoperative CEA (HR: 1.12; 95% CI: 1.03–1.22, *p* = 0.02), preoperative CA19-9 (HR: 1.09; 95% CI: 1.01–1.18, *p* = 0.03), and tumor stage (HR: 1.30; 95% CI: 1.08–1.50, *p* = 0.01) were significant prognostic factors. The presence of lymphovascular invasion (HR: 1.42; 95% CI: 1.15–1.78, *p* < 0.001), perineural invasion (HR: 1.35; 95% CI: 1.10–1.62, *p* = 0.02), and tumor budding (HR: 1.28; 95% CI: 1.05–1.52, *p* = 0.03) were associated with increased mortality risk.

For DFS, similar trends were observed: age (HR: 1.02; 95% CI: 1.00–1.05, *p* = 0.05), ASA score (HR: 1.18; 95% CI: 1.05–1.32, *p* = 0.02), WBC count (HR: 1.04; 95% CI: 1.02–1.07, *p* = 0.03), CRP levels (HR: 1.20; 95% CI: 1.09–1.33, *p* < 0.001), preoperative CEA (HR: 1.14; 95% CI: 1.05–1.23, *p* = 0.01), and preoperative CA19-9 (HR: 1.10; 95% CI: 1.03–1.20, *p* = 0.02) were significant predictors of recurrence or metastasis within one year. T stage (HR: 1.35; 95% CI: 1.10–1.60, *p* = 0.01), lymphovascular invasion (HR: 1.50; 95% CI: 1.18–1.90, *p* < 0.001), perineural invasion (HR: 1.38; 95% CI: 1.12–1.65, *p* = 0.02), tumor budding (HR: 1.30; 95% CI: 1.10–1.55, *p* = 0.02), and tumor size (HR: 1.22; 95% CI: 1.12–1.37, *p* = 0.01) were also identified as independent prognostic factors for DFS (Table 3).

## 4. Discussion

The findings of this study provide a comparison between URC and MLRC in terms of clinical, pathological, and survival outcomes. While previous studies have established differences in recurrence risk and treatment responses between these tumor locations, the current study adds new insights into the prognostic impact of tumor biology and inflammatory markers on survival and recurrence.

Several prior studies have suggested that upper rectal tumors exhibit lower local recurrence rates and improved DFS compared to mid–lower rectal tumors, likely due to differences in mesorectal involvement and surgical margins [2,10]. The present study found that although URC demonstrated a slightly better 1-year OS and DFS than MLRC, the differences did not reach statistical significance. These results are consistent with the findings of Huang et al., who reported that while mid–lower rectal cancers tend to have worse outcomes, survival differences are more pronounced over longer follow-up periods rather than within the first year [4].

The present study found that CRP and WBC levels were significantly higher in the upper rectum group, while LVI and PNI were more frequently observed in the mid–lower rectum group. Elevated CRP and WBC have been widely recognized as markers of systemic inflammation and have been associated with poorer oncological outcomes in rectal cancer patients [5,6]. The role of systemic inflammation in promoting tumor progression and metastasis may explain why CRP was an independent predictor of both OS and DFS in this study. Similar findings were reported by Prassas et al., emphasizing that CRP and other inflammatory markers should be incorporated into prognostic models for rectal cancer [11]. Chronic inflammation plays a role in tumor progression by promoting angiogenesis, immune evasion, and metastatic spread [5,6].. Several studies have demonstrated that systemic inflammatory markers, such as CRP, neutrophil-to-lymphocyte ratio (NLR), and platelet-to-lymphocyte ratio (PLR), are associated with poor prognosis in rectal cancer [12,13]. Elevated CRP and WBC levels in the upper rectum group suggest a potential inflammatory response that may influence tumor behavior. In contrast, the higher prevalence of LVI and PNI in mid–lower rectal tumors supports the hypothesis that local tumor microenvironmental factors may contribute to aggressive tumor biology [7,14]. These findings align with previous reports indicating that inflammatory markers should be integrated into prognostic models to improve risk stratification and individualized treatment planning [15].

TRG was independently associated with both OS and DFS in our analysis, supporting its potential utility as a surrogate marker of treatment response. Notably, TRG also correlated with elevated systemic inflammatory indices, further linking immune-mediated pathways with therapy effectiveness. While postoperative CEA and CA19-9 were not predictive of recurrence in this short follow-up, their prognostic utility may increase in extended surveillance settings.

The increased frequency of LVI and PNI in MLRC aligns with reports by Langrand-Escure et al. and Chuang et al., which highlight greater perineural invasion in lower rectal tumors due to their proximity to the autonomic nerve plexus [16,17]. This study confirmed that both LVI and PNI were significant predictors of poorer OS and DFS, reinforcing their role as critical risk factors for recurrence and survival with literature [1,17].

The number of removed lymph nodes was significantly higher in the upper rectum group, a finding consistent with the work of Law et al. [18]. The lower lymph node yield in MLRC may reflect the technical challenges associated with pelvic dissection and the reduced mesorectal volume in distal tumors.

Laparoscopic surgery did not significantly impact OS or DFS (*p* = 0.14 and *p* = 0.18, respectively). While some studies suggest that laparoscopic resection is associated with improved postoperative recovery and equivalent oncologic outcomes, our findings indicate that in the early survival period, the choice of surgical approach may not be a decisive factor in predicting recurrence or survival [19].

Although this study did not find a statistically significant difference in OS and DFS between URC and MLRC, the trends observed suggest that tumor biology and inflammatory response may play a more critical role than anatomical location alone. The findings support a risk-adapted approach rather than a uniform treatment strategy based solely on tumor location. Notably, the identification of CRP, pre-op CEA, and tumor burden-related factors (T stage, tumor size, LVI, PNI, budding) as independent prognostic markers suggests that systemic and tumor-specific features should be considered in clinical decision-making.

One potential clinical implication of these findings is the re-evaluation of nCRT protocols for URC. Given that URC is more biologically similar to colon cancer and that TME may not always be necessary, selecting patients for nCRT based on tumor regression grade, inflammatory markers, and molecular profiling could improve treatment individualization [2,4]. Our study’s most notable and unique finding is the observation that URC exhibits higher levels of inflammatory markers (CRP, WBC, neutrophils, and platelets), whereas MLRC demonstrates more frequent LVI, PNI, and tumor budding. This heterogeneity underscores the distinct biological behavior of tumors arising from different rectal segments. Interestingly, despite these disparities in inflammatory status and tumor biology, there was no statistically significant difference between the two groups in one-year OS or DFS. These results highlight the importance of transitioning away from uniform treatment strategies based solely on anatomical location. Rather, we underscore the need for a personalized approach that integrates systemic inflammatory response, tumor biology, and molecular characteristics. In particular, for URC, given its closer biological resemblance to colon cancer and the potential that TME may not always be necessary, existing nCRT protocols could be re-evaluated based on TRG, inflammatory markers, and molecular profiling. Moreover, our finding that TRG is an independent prognostic factor for both OS and DFS, and that it correlates with inflammatory parameters, suggests that these features should play a more prominent role in evaluating neoadjuvant treatment response and planning postoperative surveillance. By elucidating the complex interactions among tumor biology, systemic inflammation, and treatment response, this study provides a valuable steppingstone for future investigations aiming to refine risk-adaptive treatment strategies.

Although the prognostic significance of CRP, LVI, PNI, and tumor budding is well documented in the literature, our study sheds light on how these factors are distinctly distributed between URC and MLRC, further identifying shared prognostic indicators for both tumor locations. This information highlights the importance of considering tumor biology rather than relying solely on anatomical classification when making clinical decisions.

In a broader context, our observations align with prior findings that discrepancies between URC and MLRC can become more pronounced beyond one year of follow-up [3]. Elevated CRP also parallels the larger-cohort observations of Prassas et al., underscoring the correlation between inflammatory indices and recurrence risk [11]. Moreover, the higher rates of PNI and LVI in MLRC are consistent with Chuang et al., who attribute these aggressive features to distinct tumor microenvironments in lower rectal segments [16]. By situating our findings alongside these studies, we clarify how the interplay of tumor location, systemic inflammation, and pathology converges to influence patient outcomes.

In light of these prognostic insights, we propose several preliminary, risk-adapted strategies for clinical practice. For patients with URC exhibiting low-risk inflammatory indices (e.g., CRP < 10 mg/L, normal WBC) and T2N0 disease on imaging, direct surgery may be a viable alternative to routine nCRT. In contrast, MLRC showing T3N+ involvement, lymphovascular or perineural invasion, or tumor budding warrants standard long-course nCRT and total mesorectal excision. Postoperatively, closer surveillance (e.g., more frequent imaging) is advised for patients with high-risk features, such as persistently elevated CRP, abnormal tumor markers, or LVI/PNI/tumor budding. In addition, adjuvant chemotherapy may be shortened to three months for URC lacking T4 or margin-threatening disease, whereas MLRC with adverse pathology typically requires the full six-month regimen. Although these recommendations await confirmation in larger, prospective cohorts, they illustrate how prognostic markers might be integrated into personalized treatment pathways.

These findings generate a preliminary framework for future individualized treatment strategies. The proposed adjustments to nCRT or adjuvant therapy should be interpreted as exploratory and require prospective validation.

## 5. Limitations and Future Directions

This study has several important limitations. First, the sample size of 100 patients may have constrained the statistical power to detect certain between-group differences and raises concerns of overfitting in our multivariable models. Second, the retrospective design and one-year follow-up duration limit the assessment of long-term outcomes, particularly since most rectal cancer recurrences manifest within two to three years postoperatively. Third, although we focused on prognostic factors such as inflammatory markers and histopathological features, we did not collect or analyze detailed morbidity data, which are crucial for evaluating treatment-related complications. Fourth, single-center data may reduce the generalizability of these findings to other populations with differing clinical practices or demographic profiles. Our study did not include molecular or immunogenomic profiling (e.g., microsatellite instability, KRAS/BRAF status), which may further enhance biological stratification. Incorporating such parameters in future analyses could facilitate personalized oncologic decision-making. Prospective, multicenter trials with extended follow-up, larger cohorts, and robust molecular analyses are warranted to confirm and refine these observations.

## 6. Conclusions

This study provides a comprehensive comparison of upper and mid–lower rectal cancer, highlighting the prognostic impact of inflammatory markers, tumor burden, and pathological features on survival and recurrence. While tumor location alone was not an independent predictor of OS or DFS, factors such as CRP, LVI, PNI, and tumor budding emerged as critical determinants of patient outcomes. These findings support a personalized treatment approach based on tumor biology rather than strict anatomical classification. Further research is warranted to refine risk-adapted treatment strategies and explore the potential for molecular-based therapy selection in rectal cancer management [20].

## Figures and Tables

**Table 1:** Baseline clinical, surgical, and pathological characteristics of patients with upper and mid–lower rectal cancer.

Characteristic	Upper Rectum (n = 53)	Mid–Lower Rectum (n = 47)	*p*-Value
Age (years), Mean ± SD	59.79 ± 13.76	62.89 ± 12.48	0.36
Gender, n (%) male	28 (84.8)	24 (88.9)	0.89
ASA, n (%)			0.10
1	10 (18.9)	9 (19.1)	
2	15 (28.3)	14 (29.8)	
3	20 (37.7)	18 (38.3)	
4	8 (15.1)	6 (12.8)	
Hospital Stay Duration, Mean ± SD	9.45 ± 5.78	8.63 ± 7.23	0.40
Surgical Characteristics			
Operation Time, Mean ± SD	242.42 ± 104.67	281.11 ± 104.63	0.12
Laparoscopic Surgery, n (%)	29 (54.5)	40 (85.2)	0.08
Protective Ostomy, n (%)	35 (66.7)	40 (85.2)	0.15
Anastomosis Type, n (%) Circular	45 (84.8)	38 (81.5)	0.80
Biochemical Markers			
WBC, Mean ± SD	9510.91 ± 2835.06	5861.59 ± 2032.02	0.01
Neu, Mean ± SD	5083.33 ± 2263.16	3958.59 ± 1479.89	0.02
Leu, Mean ± SD	1414.47 ± 687.68	1200.63 ± 774.49	0.26
Hg, Mean ± SD	12.06 ± 1.45	11.95 ± 1.62	0.78
PLT, Mean ± SD	269,393.94 ± 65,671.50	221,333.33 ± 79,147.87	0.01
CRP, Mean ± SD	22.97 ± 32.29	5.16 ± 6.95	0.01
Albumin (g/L), Mean ± SD	40.32 ± 4.55	40.56 ± 4.45	0.83
Pre-op CEA (ng/mL), Mean ± SD	11.58 ± 10.5	16.97 ± 12.3	0.05
Post-op CEA (ng/mL), Mean ± SD	1.51 ± 1.43	2.16 ± 1.65	0.84
Pre-op CA19-9 (U/mL), Mean ± SD	10.33 ± 9.2	12.31 ± 11.1	0.45
Post-op CA19-9 (U/mL), Mean ± SD	5.2 ± 1.68	9.1 ± 2.4	0.12
Tumor Characteristics			
T Stage, n (%)			0.11
T2	15 (28.3)	12 (25.5)	
T3	25 (47.2)	26 (55.3)	
T4	13 (24.5)	9 (19.1)	
N Stage, n (%)			0.19
N0	25 (47.2)	14 (29.8)	
N1	18 (34.0)	20 (42.6)	
N2	10 (18.9)	13 (27.7)	
Positive Lymph Node	0.96 ± 2.17	0.94 ± 2.05	0.97
Tumor Size, Mean ± SD	5.66 ± 2.67	3.86 ± 3.11	0.02
Removed Lymph Nodes, Mean ± SD	25.09 ± 9.27	16.74 ± 7.74	0.00
LVI, n (%)	13 (24.5)	26 (55.3 )	0.97
PNI, n (%)	11 (20.8)	24 (51.1 )	0.04
Budding, n (%)	13 (24.5)	19 (40.4)	0.03
Outcomes			
TRG, n (%)			0.01
0	0 (0.0)	2 (4.3)	
1	6 (11.3)	7 (14.9)	
2	20 (37.7)	15 (31.9)	
3	27 (50.9)	23 (48.9)	
Local Recurrence, n (%)	5 (9.1)	7 (14.8)	0.77
Metastasis, n (%)	2 (3.0)	3 (7.4)	0.85
Excitus, n (%)	11 (21.2)	10 (22.2)	0.98

Mann–Whitney U test and Chi-square test we employed. ASA: American Society of Anesthesiologists; WBC: white blood cell count; Neu: neutrophil count; Leu: leukocyte count; Hg: hemoglobin; PLT: platelet count; CRP: C-reactive protein; CEA: carcinoembryonic antigen; CA19-9: carbohydrate antigen 19-9; LVI: lymphovascular invasion; PNI: perineural invasion; TRG: tumor regression grade.

**Table 2:** One-year oncological outcomes in patients with upper and mid–lower rectal cancer.

Outcome	Upper Rectum (%)	Mid–Lower Rectum (%)	*p*-Value
1-Year-OS	82.1% (95% CI: 72.3–91.9)	80.3% (95% CI: 70.1–90.5)	0.85
1-Year-DFS	78.6% (95% CI: 67.8–89.4)	73.4% (95% CI: 62.1–84.7)	0.72

Kaplan–Meier, Log-rank, and Cox Regression were employed. OS: overall survival; DFS: disease-free survival.

**Table 3:** Multivariate analysis of prognostic factors for overall survival and disease-free survival in patients with rectal cancer.

Variable	Odds Ratio (HR)	95% CI (Lower–Upper)	*p*-Value
OS			
Age	1.04	(1.01–1.08)	0.03
ASA Score	1.22	(1.10–1.38)	0.01
Laparoscopic Surgery	0.88	(0.72–1.05)	0.14
WBC	1.03	(1.01–1.06)	0.04
PLT	0.97	(0.91–1.02)	0.12
CRP	1.18	(1.08–1.29)	0.00
Albumin	0.92	(0.88–1.01)	0.06
Pre-op CEA	1.12	(1.03–1.22)	0.02
Pre-op CA19-9	1.09	(1.01–1.18)	0.03
T Stage	1.3	(1.08–1.50)	0.01
Removed Lymph Nodes	0.95	(0.91–1.00)	0.06
LVI Presence	1.42	(1.15–1.78)	0.00
PNI Presence	1.35	(1.10–1.62)	0.02
Budding	1.28	(1.05–1.52)	0.03
Tumor Size	1.2	(1.10–1.35)	0.01
TRG	1.15	(1.08–1.32)	0.001
DFS			
Age	1.02	(1.00–1.05)	0.05
ASA Score	1.18	(1.05–1.32)	0.02
Laparoscopic Surgery	0.92	(0.78–1.10)	0.18
WBC	1.04	(1.02–1.07)	0.03
PLT	0.98	(0.93–1.03)	0.10
CRP	1.2	(1.09–1.33)	0.001
Albumin	0.91	(0.86–1.00)	0.07
Pre-op CEA	1.14	(1.05–1.23)	0.01
Pre-op CA19-9	1.10	(1.03–1.20)	0.02
T Stage	1.35	(1.10–1.60)	0.01
Removed Lymph Nodes	0.97	(0.92–1.02)	0.09
LVI Presence	1.5	(1.18–1.90)	0.001
PNI Presence	1.38	(1.12–1.65)	0.02
Budding	1.3	(1.10–1.55)	0.02
Tumor Size	1.22	(1.12–1.37)	0.01
TRG	1.18	(1.10–1.34)	0.001

Cox proportional hazards regression was employed. OS: overall survival; DFS: disease-free survival; HR: hazard ratio; CI: confidence interval; ASA: American Society of Anesthesiologists; WBC: white blood cell count; PLT: platelet count; CRP: C-reactive protein; CEA: carcinoembryonic antigen; CA19-9: carbohydrate antigen 19-9; T Stage: tumor stage; LVI: lymphovascular invasion; PNI: perineural invasion; TRG: tumor regression grade.

## Data Availability

The data presented in this study are available on request from the corresponding author.
